# Clustering as a Means To Control Nitrate Respiration Efficiency and Toxicity in Escherichia coli

**DOI:** 10.1128/mBio.01832-19

**Published:** 2019-10-22

**Authors:** Suzy Bulot, Stéphane Audebert, Laetitia Pieulle, Farida Seduk, Emilie Baudelet, Leon Espinosa, Marie-Camille Pizay, Luc Camoin, Axel Magalon

**Affiliations:** a Aix-Marseille Université, CNRS, Laboratoire de Chimie Bactérienne (UMR7283), IMM, Marseille, France; b Aix-Marseille Université, Inserm, CNRS, Institut Paoli-Calmettes, CRCM, Marseille Protéomique, Marseille, France; University of California, Irvine

**Keywords:** cellular respiration, fluorescence microscopy, metalloprotein, nitric oxide

## Abstract

Respiration is a fundamental process that has to optimally respond to metabolic demand and environmental changes. We previously showed that nitrate respiration, crucial for gut colonization by enterobacteria, is controlled by polar clustering of the nitrate reductase increasing the electron flux through the complex. Here, we show that the formate dehydrogenase electron-donating complex, FdnGHI, also clusters at the cell poles under nitrate-respiring conditions. Its proximity to the nitrate reductase complex was confirmed by its identification in the interactome of the latter, which appears to be specific to the nitrate-respiring condition. Interestingly, we have identified a multiprotein complex dedicated to handle nitric oxide resulting from the enhanced activity of the electron transport chain terminated by nitrate reductase. We demonstrated that the cytoplasmic NADH-dependent nitrite reductase NirBD and the hybrid cluster protein Hcp are key contributors to regulation of the nitric oxide level during nitrate respiration. Thus, gathering of actors involved in respiration and NO homeostasis seems to be critical to balancing maximization of electron flux and the resulting toxicity.

## Abstract (precis)

Most bacteria rely on the redox activity of respiratory complexes embedded in the cytoplasmic membrane to gain energy in the form of ATP and of an electrochemical gradient established across the membrane. Nevertheless, production of harmful and toxic nitric oxide by actively growing bacteria as either an intermediate or side-product of nitrate respiration challenges how homeostasis control is exerted. Here, we show that components of the nitrate electron transport chain are clustered, likely influencing the kinetics of the process. Nitric oxide production from this respiratory chain is controlled and handled through a multiprotein complex, including detoxifying systems. These findings point to an essential role of compartmentalization of respiratory components in bacterial cell growth.

## INTRODUCTION

Cellular respiration is a fundamental process undergone within energy-transducing membranes through the redox activity of multimeric protein assemblies, so-called “respiratory complexes.” In most cases, electron transport is ensured by lipophilic molecules, quinones, serving as electron shuttles. This redox activity is coupled to the net translocation of ions such as protons across the membrane thereby establishing an electrochemical gradient, the proton motive force (PMF). Such a gradient powers the transport of molecules (such as proteins, ions, or antibiotics) and ATP synthesis but also participates in protein localization in prokaryotes (1–4).

Importantly, energy-transducing membranes show a high level of organization with heterogeneous distribution of respiratory complexes as observed across several bacterial lineages (5–9). Although electron transport in energy-transducing membranes is considered to be a kinetic process coupled to the diffusion of all reactants and in particular quinones (10), it is not clear to which extent subcellular distribution of respiratory complexes can have an influence on the overall kinetics. We previously evidenced polar clustering of nitrate reductase, NarGHI, an anaerobic respiratory complex in the gut bacterium Escherichia coli (9). Such spatial organization was shown to directly impact the electron flux of the associated respiratory chain. While dynamic localization of this complex plays an important role in controlling respiration, the mechanism by which it impacts the electron flux is not fully understood (11). Furthermore, optimizing electron flux through clustering of nitrate reductase likely leads to nitrite accumulation and nitric oxide (NO) production. Indeed, functioning of this electron transport chain in enteric bacteria such as E. coli and Salmonella enterica serovar Typhimurium is associated with NO production mainly resulting from the reduction of nitrite by nitrate reductase (12–15). As a consequence, both E. coli and *S.* Typhimurium produce a multitude of enzymes controlling NO homeostasis, the primary source of nitrosative stress (see for review, reference 16). Protein *S*-nitrosylation is a ubiquitous NO-dependent posttranslational modification of cysteine that regulates protein structure and function (see for review, reference 17). Recently, Seth et al. demonstrated that, in the absence of oxygen, NO oxidation is mainly catalyzed by the hybrid cluster protein Hcp, allowing its further reaction with thiols of a broad spectrum of proteins (18). Among Hcp-dependent *S*-nitrosylated targets are the nitrate reductase complex, multiple metabolic enzymes, and the OxyR transcription factor whose *S*-nitrosylation entails a distinct nitrosative stress regulon (19). In this context, we questioned not only how efficiency of the nitrate respiratory chain is attained through polar clustering of the nitrate reductase complex but also how NO homeostasis control is exerted.

Here, the electron-donating respiratory complex of formate dehydrogenase, FdnGHI, was shown to cluster at the poles under nitrate-respiring conditions. The interactome of the nitrate reductase complex confirmed the proximity and was evaluated under distinct metabolic conditions reported to impact its subcellular organization. All the identified partners were exclusively found when cells were grown under nitrate respiration. The proximity of two respiratory complexes provides a mechanistic explanation of the importance of subcellular organization upon quinone pool turnover. Noteworthy is the identification of a multiprotein complex dedicated to handle NO resulting from the enhanced activity of the electron transport chain terminated by nitrate reductase.

## RESULTS

### The electron-donating formate dehydrogenase clusters at the poles under nitrate-respiring conditions.

**FIG 1:**
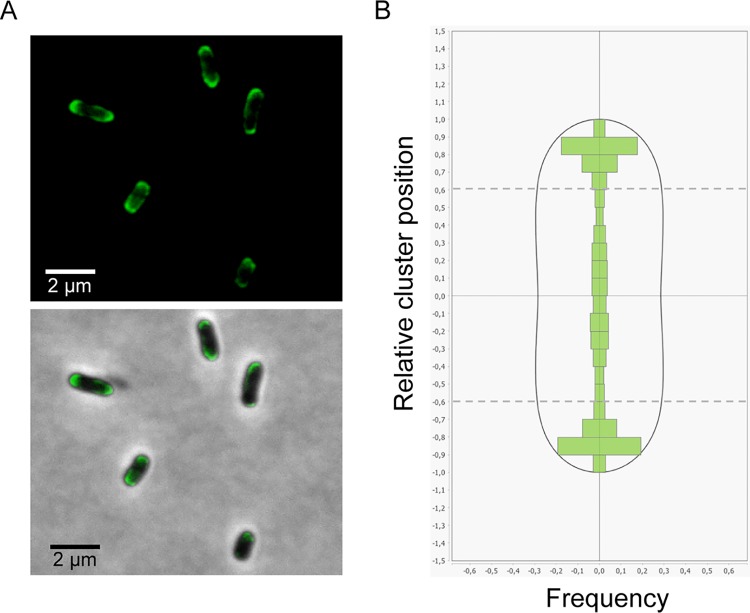
The sfGFP-labeled formate dehydrogenase complex concentrates at the cell poles under nitrate-respiring conditions. (A) Fluorescence images (top) and overlays of fluorescence and phase-contrast images (bottom) are shown for nitrate-respiring cells. (B) The histogram of the fluorescent cluster distribution across the transversal axis of nitrate-respiring LCB4215 cells is shown. Dashed lines delimit pole zones. Sixty-five percent of the clusters localize at the cell poles, and 99% of the cells exhibit polar clusters. The analysis was performed on 702 cells issued from three independent experiments. The fluorescence heat maps of the cells from each experiment are shown in Fig. S2.

As indicated above, polar clustering of the quinol-oxidizing nitrate reductase will only be beneficial to the electron flux if accompanied by quinone-reducing dehydrogenases. The electron-donating respiratory complex, formate dehydrogenase encoded by the *fdnGHI* operon, is a good candidate owing to its similar transcriptional regulation (20). Importantly, FdnGHI and NarGHI form a paradigmatic proton motive redox loop as originally postulated by Peter Mitchell in the chemiosmotic theory (21). Quinone reduction and quinol oxidation take place at different faces of the membrane and on different protein complexes, allowing net translocation of 2 protons per transferred electron (22). The E. coli formate dehydrogenase complex is composed of three subunits and organized into a physiological trimer, (FdnGHI)_3_, with a cardiolipin molecule positioned at the trimer interface (23). The C terminus of the di-heme *b*-type cytochrome subunit, FdnI, protruding from the complex in the cytoplasm was fused to superfolder green fluorescent protein (sfGFP). The genetic construct was placed at the original locus and under the control of the native promoter (see Materials and Methods). This ensures that the tagged complex is expressed in context and at physiological levels. The corresponding fusion is functional as evaluated by enzymatic activity measurements (see Fig. S1A in the supplemental material). Immunoblotting analysis using an antiserum directed against GFP demonstrated that sfGFP was fused to FdnI at the expected size with no sign of degradation (Fig. S1B). To study the spatial organization of the formate dehydrogenase complex, we visualized sfGFP distribution in live E. coli cells from exponentially growing cultures under nitrate-respiring conditions using epifluorescence microscopy. As illustrated in Fig. 1A, large patches of fluorescence are seen at cell poles. Statistical analysis of the fluorescent cluster distribution together with deduced heat maps from three independent biological replicates confirms the polar enrichment of the fusion (Fig. 1B; see Fig. S2 in the supplemental material). Therefore, formate dehydrogenase and nitrate reductase complexes tend to organize into large polar clusters.

10.1128/mBio.01832-19.1 FIG S1 The sfGFP-labeled formate dehydrogenase complex is active and stable. (A) The activity of the FdnGHI complex is unaffected by the sfGFP fusion. Formate::PMS/DCPIP oxidoreductase activity assays were performed on membrane prepared from LCB4200 (untagged version) and LCB4215 (tagged version) cells grown under nitrate-respiring conditions. The activities are expressed in μmol of CO_2_ produced min^−1^ mg^−1^ of proteins. (B) Western blot using antibodies directed against GFP shows stable expression of FdnI-sfGFP chimera. Whole-cell extract was prepared from LCB4215 cells grown under nitrate-respiring conditions to mid-exponential phase. Download FIG S1, TIF file, 0.4 MB. Copyright © 2019 Bulot et al. 2019 Bulot et al. This content is distributed under the terms of the Creative Commons Attribution 4.0 International license.

10.1128/mBio.01832-19.2 FIG S2 The sfGFP-labeled formate dehydrogenase complex concentrates at the cell poles under nitrate-respiring conditions. The heat maps built from three independent experiments are shown. From left to right, 259, 297, and 146 cells were analyzed, respectively. The mean fluorescence density is represented by a color gradient shown to the right of each heat map. Download FIG S2, TIF file, 1.4 MB. Copyright © 2019 Bulot et al. 2019 Bulot et al. This content is distributed under the terms of the Creative Commons Attribution 4.0 International license.

### The nitrate reductase complex has a specific interactome under nitrate-respiring conditions.

**FIG 2:**
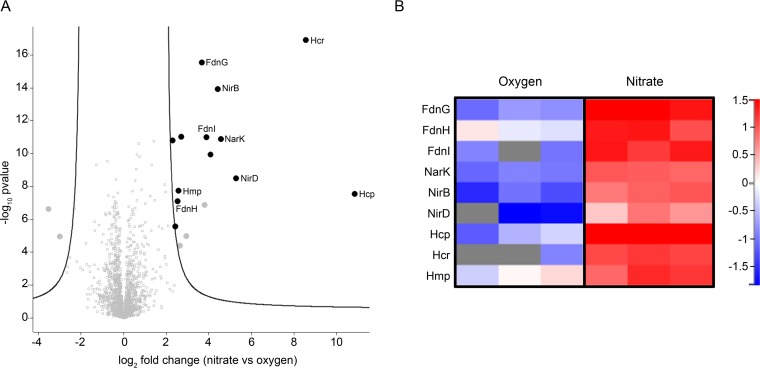
The membrane-associated nitrate reductase NarGHI has a specific interactome under nitrate-respiring conditions. (A) Volcano plot of proteins immunoprecipitated with NarG_eGFP_ in nitrate- versus oxygen-respiring conditions using nanobodies directed against GFP. Volcano plot was constructed using log_2_ fold change and absolute log_10_ of the *P* values, enabling visualization of the relationship between fold change and statistical significance, respectively. The curves show the significance threshold established from the permutation-based FDR calculations (*q* value) (see Materials and Methods). The greater the difference between the group means (i.e., the enrichment) and the greater the absolute value of *P* (i.e., the reproducibility), the more the interactors move to the top right corner of the plot. Black dots located outside the curves represent proteins differentially found between the two conditions with *q* values below 1%, while gray squares between the two curves represent proteins that do not differ between conditions. Gray dots located outside the curves represent proteins that were identified to not be specific partners of the nitrate reductase (Table S1 and Table S2). Volcano plots showing NarG_eGFP_-specific interacting partners under each condition are presented in Fig. S3. (B) Heat map focusing on formate dehydrogenase (FdnGHI), nitrate/nitrite antiporter (NarK), cytoplasmic nitrite reductase (NirBD), NO reductase/transnitrosylase (Hcp) and its redox partner (Hcr), and NO reductase (Hmp). The normalized LFQ intensities using a Z-score (means centering the variable at zero and standardizing the variance) obtained for each biological replicate under oxygen (left)- or nitrate (right)-respiring conditions are shown according to the color gradient displayed on the right. Gray represents missing values (not identified proteins).

Since the membrane-embedded nitrate reductase complex is clustered at the poles under nitrate respiration while being evenly redistributed along the cytoplasmic membrane upon aeration or anaerobic fermentation (9), we reasoned that resolving its interactome under these conditions may provide insights into the proximity with FdnGHI. To identify NarGHI partners in living cells, immunoprecipitation of NarG-eGFP (enhanced green fluorescent protein) coupled with mass spectrometry-based quantitative proteomics was performed. Importantly, chemical cross-linking has been performed to preserve transient interactions. A Student's *t* test compares the intensities of all proteins identified in replicates of the bait (i.e., GFP-tagged NarG subunit) with the intensities of all proteins identified in the control (i.e., untagged NarG subunit) (see Materials and Methods). When such analysis was performed on extracts issued from cells grown under nitrate respiration, many interactors were identified (see Fig. S3A and Table S1 in the supplemental material). Conversely, upon aeration of the culture, the main interactors were nitrate reductase structural subunits and the dedicated chaperone NarJ (Fig. S3B and Table S1). Robust statistical analysis of the data allowed the identification of interactors (*q* value under 1%) for eGFP-tagged NarG under nitrate-respiring conditions as shown in the volcano plot in Fig. 2A (see Table S2 in the supplemental material). The interactors can be grouped into two functional categories: involved in nitrate respiration or in NO homeostasis. In the first group are identified all three subunits of the electron-donating formate dehydrogenase FdnGHI (23) and the nitrate/nitrite membrane antiporter NarK (24). In the second group, the highest hits are Hcp and its associated NADH-dependent reductase Hcr (25, 26), the cytoplasmic NADH-dependent nitrite reductase complex NirBD (27, 28) and the flavohemoglobin Hmp (29). All the above-mentioned interactors were repeatedly identified in the biological replicates under nitrate-respiring conditions (Fig. 2B) as well as in the absence of formaldehyde used to capture transient interactions (see Fig. S4 in the supplemental material). Since expression of all of these interactors is upregulated by the oxygen-responsive transcriptional regulator Fnr, we conducted immunoprecipitation experiments under anaerobic conditions but without nitrate. While being detected in replicates of the bait and of the control, none of the previously identified interactors were found associated with nitrate reductase (see Fig. S3C and Table S3 in the supplemental material). Such observation makes sense as nitrite and NO are absent and entails that proximity of NirBD, Hcp, Hcr, and Hmp is linked to nitrate reduction. Interestingly, FdnGHI and NarK were also not found to be interacting with nitrate reductase complex under this condition.

10.1128/mBio.01832-19.3 FIG S3 The membrane-associated nitrate reductase NarGHI has a specific interactome under nitrate-respiring conditions. (A and B) Volcano plots of proteins immunoprecipitated with NarG_eGFP_ versus the untagged version of NarG under nitrate-respiring (A) or oxic (B) conditions. The volcano plot was constructed and displayed as described in the legend to Fig. 2. Black dots located outside the curves represent proteins differentially found between the two conditions with *q* values below 1% (Table S1). (C) Partners of the nitrate reductase under nitrate-respiring conditions no longer interact with the complex in anoxic fermentation. Shown is a heat map focusing on the 9 proteins identified in Fig. 2 displaying the Z-scored LFQ intensity obtained for each biological replicate using untagged (left) or tagged (right) version of the nitrate reductase under anoxic fermentative conditions (Table S3). Gray represents missing values (not identified proteins). Download FIG S3, TIF file, 1.8 MB. Copyright © 2019 Bulot et al. 2019 Bulot et al. This content is distributed under the terms of the Creative Commons Attribution 4.0 International license.

10.1128/mBio.01832-19.4 FIG S4 A similar nitrate reductase interactome is obtained in the absence of cross-linking. The experiment was done exactly as for Fig. S3A, but the cross-linking step was avoided. The volcano plot of proteins immunoprecipitated with NarG_eGFP_ versus the untagged version of NarG under nitrate-respiring conditions is shown. The volcano plot was constructed and displayed as described in the legend to Fig. 2, except the threshold was fixed by an enrichment greater than 4-fold and a *P* value of <0.05. Proteins identified in the legend to Fig. 2 are represented by labeled black dots. Download FIG S4, TIF file, 2.1 MB. Copyright © 2019 Bulot et al. 2019 Bulot et al. This content is distributed under the terms of the Creative Commons Attribution 4.0 International license.

10.1128/mBio.01832-19.6 TABLE S1 List of identified proteins immunoprecipitated with NarG_eGFP_ versus untagged version of NarG under nitrate-respiring (Fig. S3A) or oxic (Fig. S3B) conditions. Download Table S1, XLSX file, 0.01 MB. Copyright © 2019 Bulot et al. 2019 Bulot et al. This content is distributed under the terms of the Creative Commons Attribution 4.0 International license.

10.1128/mBio.01832-19.7 TABLE S2 List of identified proteins immunoprecipitated with NarG_eGFP_ under nitrate- versus oxygen-respiring conditions (Fig. 2). Download Table S2, XLSX file, 0.01 MB. Copyright © 2019 Bulot et al. 2019 Bulot et al. This content is distributed under the terms of the Creative Commons Attribution 4.0 International license.

10.1128/mBio.01832-19.8 TABLE S3 List of LFQ intensities for all identified partners of NarG_eGFP_ and full data set. Download Table S3, XLSX file, 1.7 MB. Copyright © 2019 Bulot et al. 2019 Bulot et al. This content is distributed under the terms of the Creative Commons Attribution 4.0 International license.

**FIG 3:**
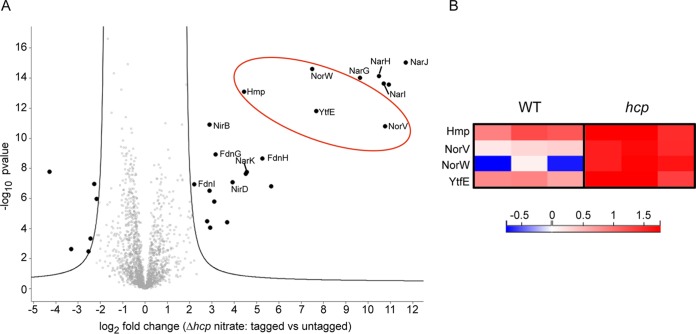
Interactome of the nitrate reductase in the *hcp*-deficient strain. (A) Volcano plot of proteins immunoprecipitated with NarG_eGFP_ versus untagged version of NarG under nitrate-respiring conditions. The volcano plot was constructed and displayed as described in the legend to Fig. 2. Black dots located outside the curves represent proteins differentially found between the two conditions with *q* values below 1% (Table S4). Proteins more enriched using the *hcp* strain are circled in red. (B) The two NO reductases, Hmp and NorV with its redox partner NorW, and YtfE (RIC) protein were found more abundantly associated with the nitrate reductase in the *hcp* strain. The heat map shows the Z-scored LFQ intensities obtained for each biological replicate in the wild-type strain (left) and in the *hcp* strain (right) grown under nitrate-respiring conditions according to the color gradient below.

Nitrate respiration being associated with NO production and as such prone to Hcp-dependent *S*-nitrosylation of most of the identified partners of NarG subunit, we questioned whether such posttranslational modification can influence protein-protein interactions. Indeed, *S*-nitrosylation of Hcp was shown to promote conformational changes responsible for protein interaction with its targets among which nitrate reductase, formate dehydrogenase, and NirBD complexes (18). To evaluate such a possibility, we conducted interactome studies in an *hcp* strain under nitrate-respiring conditions. We confirmed that polar clustering of eGFP-tagged nitrate reductase was not affected by the absence of Hcp (see Fig. S5 in the supplemental material). The interactome comprised all previously identified partners (Fig. 3A; see Table S4 in the supplemental material). Notably, it includes three additional proteins involved in NO homeostasis, the flavorubredoxin NorV and its NADH-dependent reductase, NorW (30, 31), as well as the diiron protein YtfE (32, 33). Thus, Hcp-dependent *S*-nitrosylation of targets is not responsible for the identified interactome of the nitrate reductase complex comprising stable components such as formate dehydrogenase, NarK or NirBD, and others, the nature of which depends on the level of nitrosative stress (Fig. 3B). Indeed, it is anticipated that the absence of Hcp entails higher nitrosative stress since the *norVW* genes are only expressed under conditions of high NO concentration (34, 35).

10.1128/mBio.01832-19.5 FIG S5 The subcellular organization of the nitrate reductase is unchanged in the *hcp*-deficient strain. (A) Fluorescence images (top) and overlays of fluorescence and phase-contrast images (bottom) are shown for nitrate-respiring cells. (B) The histogram of the fluorescent cluster distribution across the transversal axis of nitrate respiring LCB3063 cells harboring pVA70GFP is shown. Dashed lines delimit pole zones. The analysis was performed on 797 cells issued from three independent experiments. (C) The heat map shows the mean fluorescent profiles from 797 cells. The fluorescence density is represented by a color gradient shown on the right side. Download FIG S5, TIF file, 1.6 MB. Copyright © 2019 Bulot et al. 2019 Bulot et al. This content is distributed under the terms of the Creative Commons Attribution 4.0 International license.

10.1128/mBio.01832-19.9 TABLE S4 List of identified proteins immunoprecipitated with NarG_eGFP_ versus the untagged version of NarG under the nitrate-respiring condition but in the absence of *hcp* (Fig. 3). Download Table S4, XLSX file, 0.01 MB. Copyright © 2019 Bulot et al. 2019 Bulot et al. This content is distributed under the terms of the Creative Commons Attribution 4.0 International license.

### Hcp and NirBD play nonredundant major roles during nitrate respiration.

**FIG 4:**
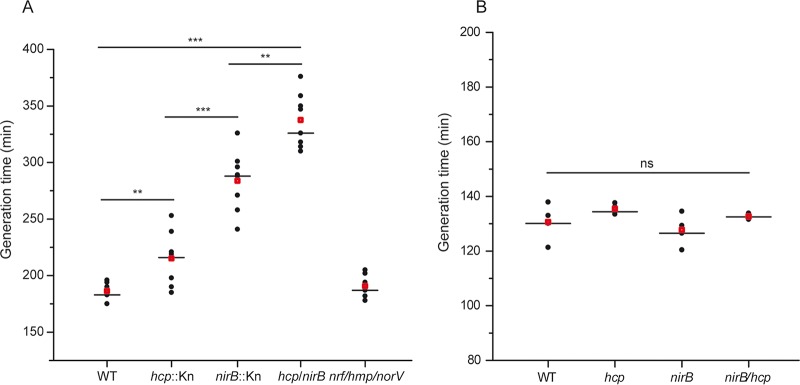
Inactivation of *nirB* and *hcp* induces a growth defect under nitrate-respiring conditions caused by elevated nitrosative stress. Generation times of strains JCB4011 (WT), LCB4136 (*hcp*), LCB4121 (*nirB*), LCB4137 (*nirB hcp*) grown under anaerobiosis in defined medium with glycerol as sole carbon source and nitrate as the sole electron acceptor without Casamino Acids (A) or supplemented with BCAA (B) are shown. Eight (A) and four (B) independent assays are, respectively, shown. Medians are represented by a horizontal line and averages by a red square. Statistical significance between the different strains was calculated using the Mann-Whitney nonparametric test: ***, *P < *0.001; **, 0.001 < *P < *0.01; ns, not significant (*P > *0.05).

Clustering of quinone-reducing and quinol-oxidizing respiratory complexes is expected to maximize turnover of the quinone pool and nitrite accumulation further reduced into NO by NarGHI. It is thus important to question the relative contribution of the identified nitrate reductase partners to NO homeostasis. While proximity of NirBD and NarK would minimize the availability of nitrite, two distinct protective roles have been associated with Hcp during nitrosative stress, NO reduction and *S*-nitrosylation. Thanks to the use of a strain that lacks both nitrite reductases (NirB and NrfA) and other known NO reductases, NorVW and Hmp, Wang et al. demonstrated that Hcp is a high-affinity NO reductase (26). Only in this background strain, was a clear growth defect phenotype associated with *hcp* deletion during nitrate respiration. However, Seth et al. reported that single deletion of *hcp* greatly suppresses *S*-nitrosylation (18). IlvD, the dihydroxy-acid dehydratase required for branched-chain amino acid (BCAA) biosynthesis, is a major target for *S*-nitrosylation associated with enzyme inactivation in E. coli (36, 37). NO exposure induces transient BCAA auxotrophy thanks to the combined action of detoxifying and repair systems. To ascertain the relative contribution of Hcp and NirBD to NO homeostasis during nitrate respiration, growth of the individual mutant strains was performed in minimal medium in the absence of amino acids. A severe growth defect was observed upon *nirB* deletion and to a lesser extent with *hcp* (Fig. 4A). Notably, the double mutant strain shows an even more pronounced impact indicating that both systems play nonredundant roles. We additionally observed that simultaneous deletion of *nrf*, *hmp*, and *norV* has no impact on growth. Upon BCAA addition, all mutant strains behave similarly to the wild type (Fig. 4B). Taken together, our findings demonstrate unequivocally that NirBD and Hcp are key contributors to regulation of the NO level during nitrate respiration.

## DISCUSSION

Respiration is a central feature for most prokaryotes to harvest energy. However, it is unclear how components of a respiratory chain are organized to achieve high electron transport kinetics while minimizing production of harmful and toxic reactive oxygen or nitrogen species. Here, we describe the spatial clustering of two distinct respiratory complexes at the cell poles surmising an impact on the electron flux of the electron transport chain. Moreover, a multiprotein complex is functionally associated with control of NO production resulting from the activity of this clustered electron transport chain.

The impacts of compartmentalization on metabolic pathways have been the subject of intense studies during several decades, bolstered by the recognition that spatial heterogeneity is a hallmark of living organisms (see for review, references [Bibr B38][Bibr B39][Bibr B42]). Clustering of multiple consecutive enzymes in a metabolic pathway proved to accelerate processing of intermediates (43–45). Similarly, energy-transducing membranes are considered a compartmentalized fluid with both lipid and protein nanodomains (46–48). Supramolecular assemblies of distinct respiratory complexes have been reported in all branches of life (49), suggesting that they confer a kinetic advantage by trapping or channeling quinone and/or cytochrome *c*, which serve as electron shuttles (50, 51). However, kinetic experiments argued against such pool behavior, whereby electron shuttles can be compartmentalized within supercomplexes (52, 53). Heterogeneous organization of respiratory complexes in the form of supercomplexes might in turn facilitate diffusion of quinones as deduced by Monte Carlo simulations (54). Here, we provide evidence that formate dehydrogenase and nitrate reductase complexes concentrate at the cell poles under nitrate-respiring conditions and may even be in close proximity, as revealed by immunoprecipitation experiments. Notably, among the other electron-donating dehydrogenases produced under this condition, FdnGHI is the unique complex identified in the interactome. In support of this apparent selectivity, several reports have indicated their copurification over a wide range of solubilization conditions (55, 56). According to the metabolite microdomain hypothesis (43–45), the probability of quinones being processed in such domains at the poles is expected to be higher than a random-distribution condition. The same might hold true within segregated clusters of respiratory complexes composed of several tens of the same complex and involved in aerobic respiratory chains (8). As such, the tendency of respiratory complexes to cluster might have the effect of organizing the membrane into dynamic compartments within which distinct accessibility to the quinone pool is questioned. Quinone diffusion between respiratory complexes is considered to be the rate-limiting step for the mitochondrial and bacterial electron transport chains (57, 58). So far, diffusion of fluorescently labeled quinones was shown to be homogeneous in live bacterial cells as assessed by FRAP (fluorescence recovery after photobleaching) monitoring the dynamics of an ensemble of molecules, a method weakly sensitive to separate subpopulations with different diffusion characteristics (8). More interesting is that proximity of formate dehydrogenase and nitrate reductase seems to be modulated by cellular cues, as deduced from the lack of interaction under specific metabolic conditions. It is worth mentioning the anionic lipids, among which cardiolipin accumulates at the poles and septa of the rod-shaped bacterium (59, 60) and was shown to interact with both complexes (23, 61). Due to the dynamic localization profile of NarGHI, cardiolipin can be discarded as a cue for polar clustering of this complex. However, its importance for FdnGHI oligomerization and/or localization as evidenced for the osmosensing transporter ProP (62) remains open and awaits further studies. One immediate consequence of clustering both respiratory complexes is very likely an increase of the electron flux and the accumulation of nitrite further reduced to NO by NarGHI.

During nitrate respiration, a set of potent interactors have been identified, all of which are associated with nitrate utilization. The activities of NirBD and NarK within this multiprotein complex would limit the availability of toxic nitrite and thus its further reduction to NO by NarG. However, it has long been reported that nitrite accumulation under this growth condition leads to nitrosative stress, as confirmed here with the detection of a set of NO-detoxifying enzymes. In addition, the severe growth defect observed in the *nirBD* strain is fully reverted upon BCAA addition indicative of the inactivation of the Fe-S cluster-dependent IlvD enzyme, known as the crucial target for NO-induced bacteriostasis (36, 37). Thus, our findings provide the first direct evidence for a role in NO detoxification by Nir. BCAA supplementation has also been reported to confer resistance to NO without increasing NO consumption in both E. coli and *S.* Typhimurium (36, 63). It is well established that under aerobic or microaerobic conditions, NO has a relatively short half-life primarily due to auto-oxidation by O_2_ and dioxygenation by Hmp, being the dominant detoxification system at O_2_ concentrations as low as 35 μM (64). Under our experimental conditions, it is unlikely that Hmp, one of the interactors, provides adequate protection in the absence of oxygen (29). Conversely, Hcp has recently been recognized as being responsible for *S*-nitrosylation of multiple targets, among which are OxyR and the nitrate reductase complex (18). Furthermore, while Hcp-dependent *S*-nitrosylation was reported to inhibit NarGHI (18), this complex is likely not the main target affected by NO stress, as evidenced by complete growth recovery of mutant strains displaying NO stress upon BCAA addition. Hcp protein is present in most bacteria performing nitrate ammonification, such as enterobacteria, but absent from denitrifiers. While Hcp is responsible for *S*-nitrosylation, Wang et al. reported an NO-detoxifying activity thanks to its ability to reduce NO to N_2_O (26). Interestingly, both activities critically depend on the interaction with the NADH-dependent Hcp reductase, Hcr (25), and the NAD^+^/NADH ratio. Under a high-metabolic regimen associated with a high NAD^+^/NADH ratio, SNO synthase activity of Hcp will be favored. On the contrary, a low NAD^+^/NADH ratio will facilitate NADH-dependent reduction of NO by the Hcp-Hcr complex. Thus, our finding is consistent with a distinct role for Hcp according to the metabolic regime. We validated the importance of Hcp by observing a growth defect associated with the *hcp* strain while being reversed upon BCAA addition.

Altogether, our findings demonstrate that during nitrate respiration, (i) formate dehydrogenase coclusters with nitrate reductase at the cell poles, (ii) a multiprotein complex is formed and dedicated to handle NO production resulting from the enhanced activity of nitrate reductase, and (iii) NirBD and Hcp are the major nonredundant actors to control an endogenous NO source. Our study opens the possibility to investigate the effects of microcompartmentalization of respiratory complexes on quinone pool turnover, such as through the use of mathematical modeling approaches but also by questioning the respective roles of FdnGHI and NarGHI in their polar clustering.

## MATERIALS AND METHODS

### Bacterial strains and growth conditions.

The E. coli strains and plasmids used in this study are described in Table S5 in the supplemental material (9, 65, 66). E. coli strains were grown in LB medium or on LB agar plates for genetic construction, transformation, and storage. For the various assays, bacteria were grown aerobically at 37°C in a defined medium supplemented with 140 mM glycerol used as the main carbon source and 100 mM nitrate. Anaerobic growth of bacteria is performed in gas-tight Hungate tubes under Ar atmosphere. For anoxic fermentative growth, glycerol was replaced by 40 mM glucose and nitrate was omitted. The defined medium is composed of potassium phosphate buffer (100 mM) adjusted to pH 7.4, ammonium sulfate (6 mM), NaCl (9 mM), magnesium sulfate (2 mM), sodium molybdate (5 μM), sodium selenite (1 μM), Mohr’s salt (10 μM), calcium chloride (100 μM), Casamino Acids (0.5%), and thiamine (0.01%). Antibiotic was added when required. When specified, Casamino Acids were replaced by isoleucine (400 μM), leucine (800 μM), and valine (600 μM), or not added.

10.1128/mBio.01832-19.10 TABLE S5 Strain, plasmid, and oligonucleotide list. Download Table S5, DOCX file, 0.02 MB. Copyright © 2019 Bulot et al. 2019 Bulot et al. This content is distributed under the terms of the Creative Commons Attribution 4.0 International license.

P1 transduction was used to transfer the *hcp*::*kn*, *nirB*::*kn*, *norV*::*kn*, *hmp*::*kn*, *nrfA*::*kn*, and *fdoG*::*kn* mutations from corresponding Keio collection strains (67) into the JCB4011 strain (68). The transductants were purified twice on LB plates supplemented with kanamycin (30 mg/liter). The kanamycin cartridge was eliminated using pCP20 plasmid (69). Mutant genotypes were verified by PCR amplification using primers flanking the *hcp*, *nirB*, *norV*, *hmp*, *nrfA*, and *fdoG* genes [*nirB*(fwd)/*nirB*(rev), *hcp*(fwd)/*hcp*(rev), *norV*(fwd)/*norV*(rev), *hmp*(fwd)/*hmp*(rev), *nrfA*(fwd)/*nrfA*(rev) and *fdoG*(fwd)/*fdoG*(rev)].

The *fdoG*-deficient strain LCB4200 was used as recipient for integration of the translational *fdnI-sfgfp* fusion at the natural chromosomal locus using λ-red mediated recombination method as described in reference 70. *sfgfp* was amplified from plasmid pSWU19 *sfgfp-linker-frzX* (kindly provided by T. Mignot’s lab, Laboratoire de Chimie Bactérienne, France) using primers *sfgfp*(fwd) and *sfgfp*(rev). The kanamycin resistance gene (*kn*) was amplified from genomic DNA purified from a Keio collection strain using primers *kn*(fwd) and *kn*(rev). The ′*fdnI-sfgfp*::*kn* fragment used for the recombination was amplified from the above-described *sfgfp* and the *kn* fragments using primers ′*fdnI*-*sfgfp*(fwd) and *kn*(rev), which imparted flanking homologous regions to the chromosomal *fdnGHI* operon. The kanamycin-resistant recombinant LCB4215 was purified twice on LB plates supplemented with kanamycin (30 mg/liter) and characterized by PCR using primers *fdnI*(fwd) and *fdnI*(rev) and sequencing. The oligonucleotides used in this study are described in Table S5 (9, 65, 66).

### Immunoprecipitation of the nitrate reductase.

Immunoprecipitation was done with ChromoTek GFP-Trap on membrane extracts issued from the nitrate reductase-deficient strain JCB4023 (or the LCB4174 strain deficient for *hcp*) harboring the pVA70GFP plasmid carrying the *narG_egfp_HJI* operon under the control of its native promoter. The plasmid pVA70 containing the untagged version of the operon was used as a negative control. Each strain was grown overnight in 5 ml of defined medium containing either glycerol and nitrate (aerobic and nitrate-respiring conditions) or glucose (fermentative conditions) in 50-ml conical flasks and subcultured the next day into 30 ml of identical medium in 500-ml conical flasks at 37°C under 180 rpm. At mid-exponential phase (optical density at 600 nm [OD_600_] of 0.9), the cultures corresponding to nitrate-respiring and fermentative conditions were transferred in gas-tight bottles, gassed with argon, and incubated during an additional hour at 37°C. This procedure ensures optimal polar localization of the nitrate reductase under nitrate-respiring conditions while resulting in an even distribution of the complex under anoxic fermentative ones (9). To preserve potential transient interactions, polar clustering being oxygen sensitive, experiments were performed in the presence of 1% formaldehyde added to the cell culture when stated. After 30 min of incubation at room temperature, the cross-linking reaction was stopped by adding 125 mM glycine and the reaction mixture was incubated for a further 15 min at room temperature before proceeding to three washing steps in a buffer containing 150 mM Tris-HCl (pH 7) and 1 mM MgCl_2_. The cultures corresponding to aerobic conditions were stopped at an OD_600_ of 1.2 and subjected to the same treatment. Membrane fractions were prepared from each culture and solubilized overnight at 4°C with a combination of 1% Triton X-100 and 1% *n*-dodecyl β-d-maltoside (DDM). The immunoprecipitation was done by following the supplier’s recommendations (ChromoTek). The immunoprecipitate samples were controlled on silver nitrate-stained 10% SDS–polyacrylamide gel prior to analysis by mass spectrometry.

### Mass spectrometry and data analysis.

Each condition was injected in 3 biological replicates and 2 technical replicates in liquid chromatography (Ultimate 3000 RSLCnano chromatography system [Thermo Fisher Scientific]) coupled with an Orbitrap Fusion Lumos Tribrid mass spectrometer (Thermo Fisher Scientific). First, peptides were concentrated and purified on a precolumn from Dionex (C_18_ PepMap100, 2-cm by 100-μm inside diameter [i.d.], 100-Å pore size, 5-μm particle size) in solvent A (0.1% formic acid in 2% acetonitrile). In the second step, peptides were separated on a reverse-phase LC EASY-Spray C_18_ column from Dionex (PepMap RSLC C_18_, 50 cm by 75-μm i.d., 100-Å pore size, 2-μm particle size) at a flow rate of 300 nl/min and 40°C. After column equilibration using 4% of solvent B (20% water, 80% acetonitrile, 0.1% formic acid), peptides were eluted from the analytical column by a two-step linear gradient (4 to 20% acetonitrile–H_2_O–0.1% formic acid for 220 min and 20 to 45% acetonitrile–H_2_O–0.1% formic acid for 20 min). For peptide ionization in the EASY-Spray nanosource, the spray voltage was set at 2.2 kV and the capillary temperature was set at 275°C. The mass spectrometer was used in the data-dependent mode to switch consistently between mass spectrometry (MS) and tandem MS (MS/MS). The time between master scans was set to 3 s. MS spectra were acquired with the Orbitrap in the *m*/*z* range of 375 to 1,500 at a full width at half maximum (FWHM) resolution of 60,000 measured at *m*/*z* 200. The AGC target was set at 4.0 × 10^5^ with a 50-ms maximum injection time. The more abundant precursor ions were selected, and collision-induced dissociation fragmentation at 35% was performed and analyzed in the ion trap using the “Inject Ions for All Available Parallelizable time” option with a maximum injection time of 105 ms and an AGC target of 1.0 × 10^5^. Charge state screening was enabled to include precursors with 2 and 7 charge states. Dynamic exclusion was enabled with a repeat count of 1 and a duration of 60 s. These chromatographic conditions were previously optimized with a protein pool from all the samples.

### Quantitative proteomics processing.

For data processing, we used the free suite MaxQuant version 1.6.1.0 (71). The relative intensities based on label-free quantification (LFQ) were calculated using the MaxLFQ algorithm (72). The liquid chromatography (LC)-MS raw acquisitions were processed by the Andromeda search engine integrated into MaxQuant (71). The identification of the precursor ions present in the mass spectra was performed by comparison with the protein database of E. coli (organism ID 83333) extracted from UniProt on 20 November 2017 and containing 4,306 entries. This database was supplemented with a set of 245 proteins that are commonly found as contaminants. The following parameters were used for this search: (i) trypsin cleavage authorization before prolines, (ii) authorization of two failed cleavages, (iii) fixed modification of cysteines by carbamidomethylation (+57.02146 Da) and variable modification of methionine by oxidation (+15.99491) and N-terminal proteins by acetylation (+42.0116), (iv) authorization of 5 modifications per peptide, and (v) a minimum peptide length of 7 amino acids and a maximum mass of 4,600 Da. Spectral alignment was performed in two dimensions: the elution time of the precursor ions (min) and the mass over charge (*m*/*z* [amu]). The “Match between runs” option has been enabled to allow the transfer of identifications between LC-MS/MS based on the mass and the retention time using the default settings. The false-positive rate on identification was set at 1% at the peptide and protein levels to define the list of identified proteins. The statistical analysis was carried out with the Perseus program (version 1.6.1.2) in the MaxQuant environment. The normalized intensity LFQ was transformed by a base logarithm of 2 to obtain a normal distribution. Differential protein expressions were evidenced by the application of a Student's *t* test associated with a permutation-based false-discovery rate (pFDR [*q* value]) at 1% using 250 permutations. The differential proteomics analysis was carried out on identified proteins after removal of proteins only identified with modified peptides and proteins from the contaminant database. Peptides shared with other proteins were not included to calculate the LFQ intensities to avoid biased protein quantification. Finally, to improve quantification of correctly identified proteins, only proteins that were represented at 70% under at least one condition were kept.

### Cell extract preparation.

Bacterial cells grown under nitrate-respiring conditions were harvested at mid-exponential phase, washed, and resuspended in a buffer containing 40 mM Tris-HCl (pH 7.4) and 1 mM MgCl_2_. Bacterial cells were broken by one passage through a French press. After an initial centrifugation at 28,000 × *g*, supernatants consisting of a clear whole-cell extract were collected and further submitted to an ultracentrifugation at 380,000 × *g* allowing the isolation of membrane vesicles.

### Enzyme activity and protein quantification.

Formate dehydrogenase activity was measured by 2,6-dichlorophenolindophenol (DCPIP) reduction mediated by phenazine methosulfate (PMS) as described previously (73). The cuvette was filled with 3.5 ml of 25 mM phosphate buffer (pH 6.5) gassed with argon, 0.137 mM DCPIP, 0.28 μM freshly dissolved PMS, and 17 to 70 μg of membrane proteins. The endogenous rate of reduction of PMS was measured at 600 nm during 1 to 2 min before the reaction was initiated by the addition of 17 mM formate. Upon subtraction of the endogenous rate, the enzymatic activity was calculated using an extinction coefficient of 20 mM^−1^ cm^−1^. The total protein concentration was measured using bovine serum albumin (Sigma) as the standard as described in reference 74.

### Western blot assay.

Immunoblotting was performed using antibodies raised against GFP (ChromoTek) on whole-cell extracts run on 10% SDS–polyacrylamide gel and transferred on nitrocellulose membrane.

### Fluorescence microscopy.

Bacterial cells were grown at 37°C under nitrate-respiring conditions up to the mid-exponential phase. Two microliters of the suspension was mounted on microscope slides covered by a thick fresh agar pad and imaged as described in reference 9. Imaging was performed using a Nikon Eclipse TiE PFS inverted epifluorescence microscope (100× oil objective, NA 1.3) and a Hamamatsu Orca Flash LT 4.0 sCMOS camera.

### Image and statistical analysis.

All image analysis and statistical representations of fluorescence were performed with FIJI and MicrobeJ softwares (75, 76). The distribution of fluorescent clusters was obtained by a local maxima detection algorithm and reported to the relative longitudinal axis position; the signal was prefiltered by band-pass fast Fourier transform (FFT). The average distribution heat maps of FdnI-sfGFP were obtained by the projection of the raw fluorescence of all cells in a group. Each cell shape and the associated fluorescence signal were previously morphed to the group mean shape using MicrobeJ v5.13k.

### Data availability.

The mass spectrometry proteomics data, including search result, have been deposited in the ProteomeXchange Consortium (www.proteomexchange.org) via the PRIDE partner repository with data set identifier PXD012320.
