# Supplementary figures and images for: Clustering as a Means To Control Nitrate Respiration Efficiency and Toxicity in Escherichia coli
Source: mBio. 2019 Oct 22;10(5):e01832-19. doi: 10.1128/mBio.01832-19 (PMC6805990; doi:10.1128/mBio.01832-19)

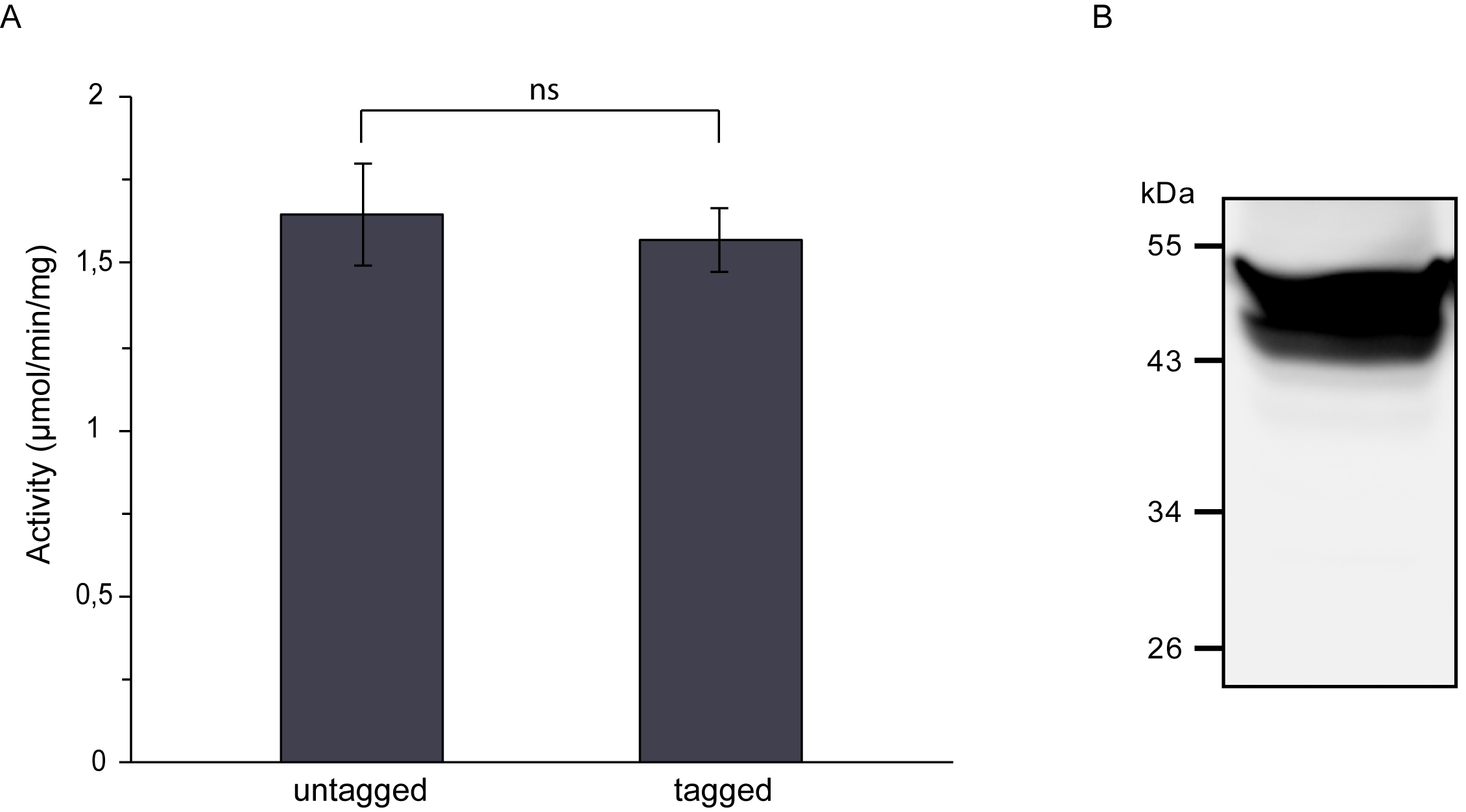

Supplement: FIG S1 [file mBio.01832-19-sf001.tif]

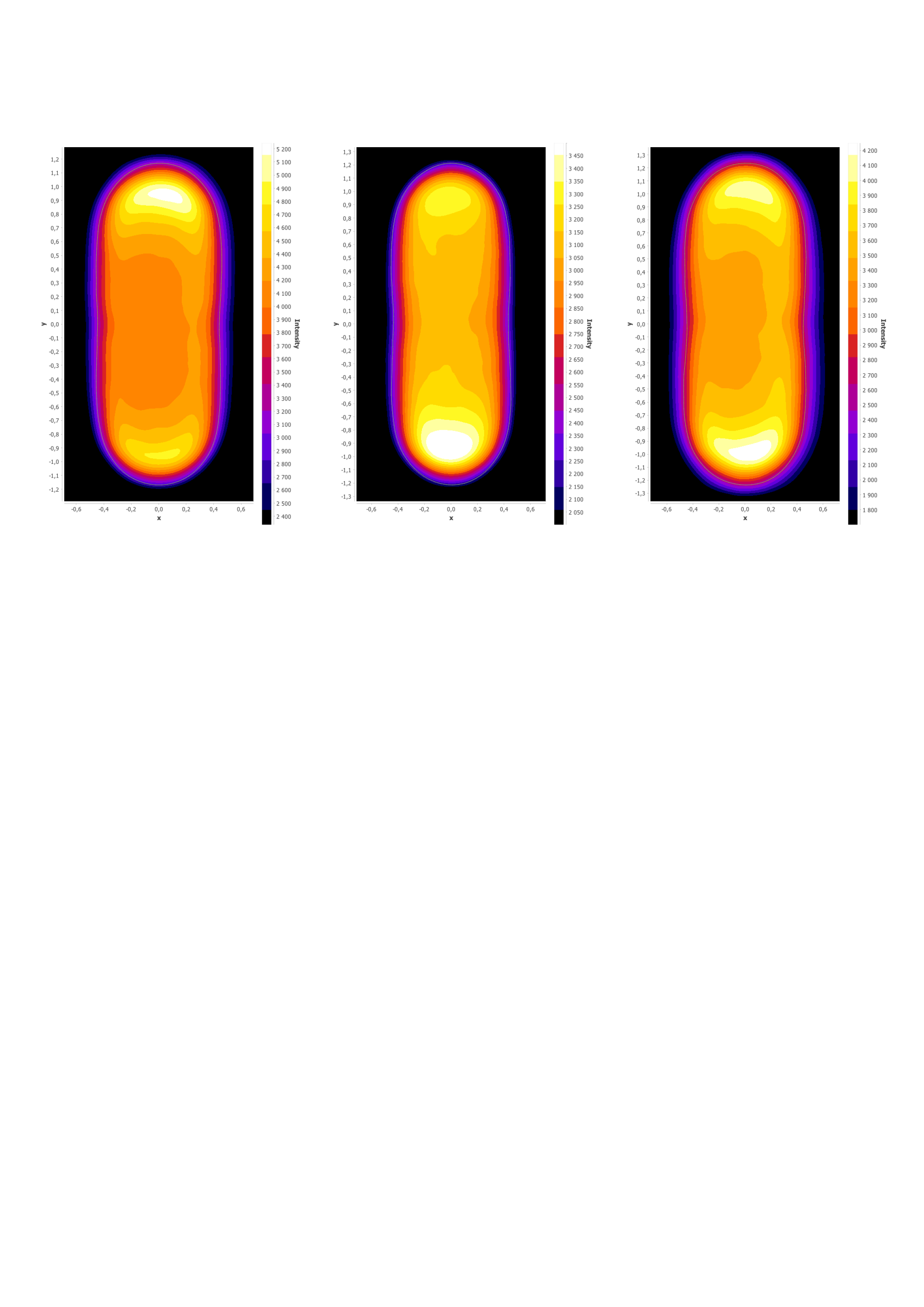

Supplement: FIG S2 [file mBio.01832-19-sf002.tif]

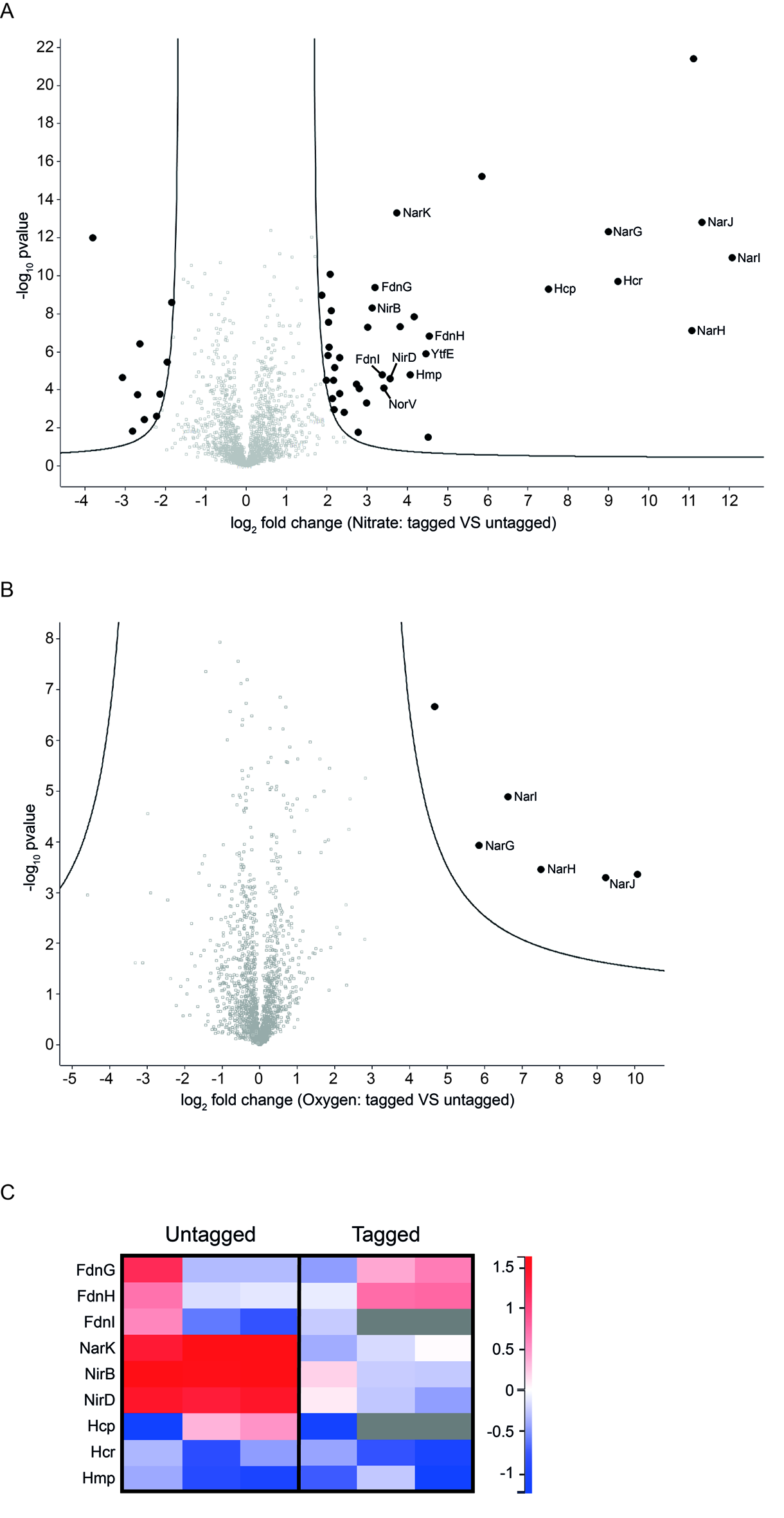

Supplement: FIG S3 [file mBio.01832-19-sf003.tif]

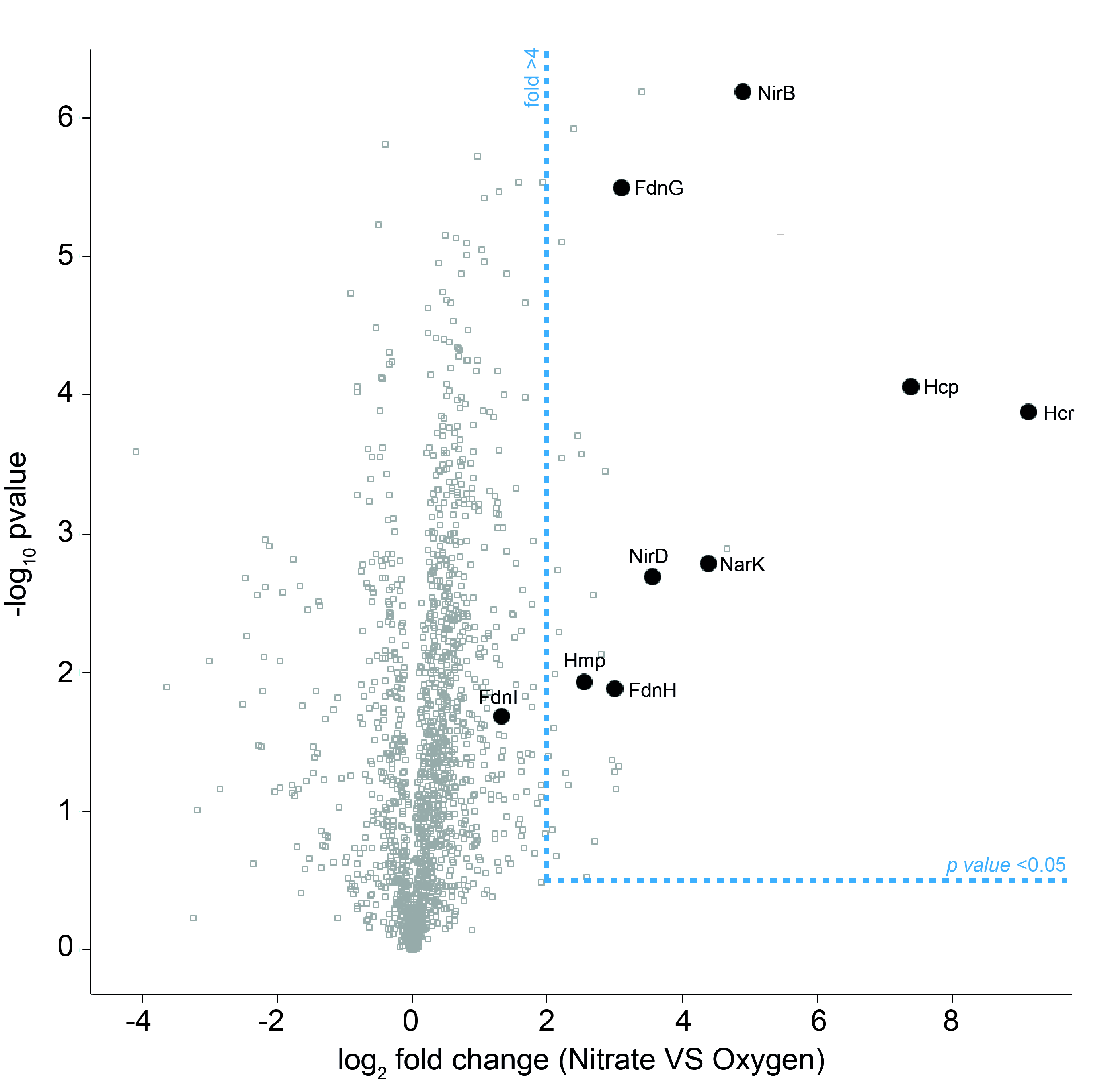

Supplement: FIG S4 [file mBio.01832-19-sf004.tif]

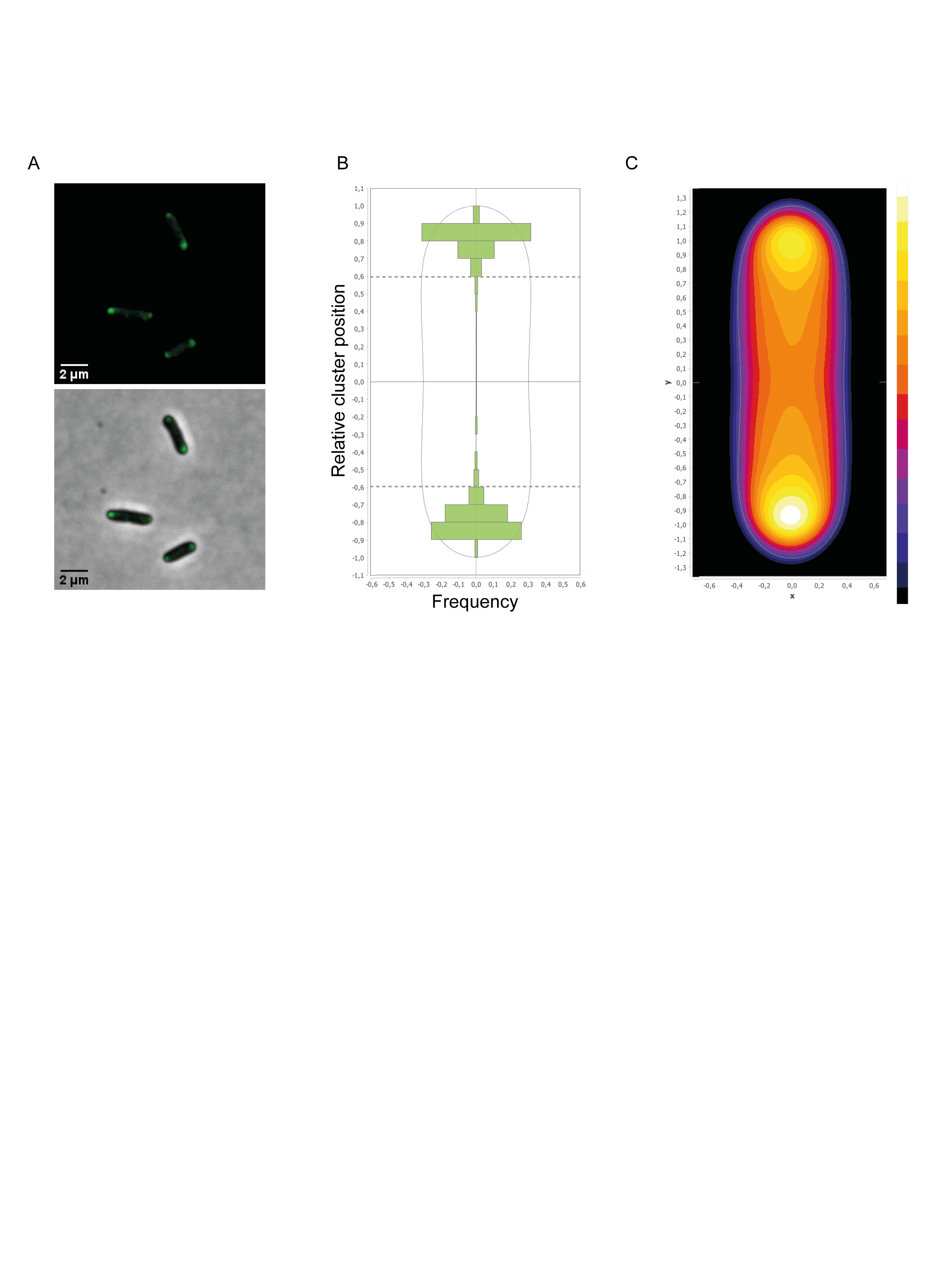

Supplement: FIG S5 [file mBio.01832-19-sf005.tif]
