# Supplementary material for: Clustering as a Means To Control Nitrate Respiration Efficiency and Toxicity in Escherichia coli
Source: mBio. 2019 Oct 22;10(5):e01832-19. doi: 10.1128/mBio.01832-19 (PMC6805990; doi:10.1128/mBio.01832-19)
Supplement: TABLE S5 [file mBio.01832-19-st005.docx]

**Table S5. Strain, plasmid and oligonucleotide list**

| Strain/Plasmid | Relevant genotype/Description | Reference |
| --- | --- | --- |
| JCB4011 | RK4353, *∆napA-B*, *∆narZ::*Ω, Spc^R^ | (65) |
| JCB4023 | RK5353, ∆*napA-B*, ∆*narZ::*Ω, *narG::ery*, Spc^R^ | (65) |
| LCB4174 | JCB4023, *hcp::kn* | This work |
| LCB4121 | JCB4011, ∆*nirB* | This work |
| LCB4136 | JCB4011, ∆*hcp* | This work |
| LCB4137 | JCB4011, *∆nirB*, *∆hcp* | This work |
| LCB4312 | JCB4011, *ΔnorV Δhmp nfrA ::kn* | This work |
| LCB4200 | JCB4011, ∆*fdoG* | This work |
| LCB4215 | JCB4011, ∆*fdoG*, *fdnI-sfgfp*, Kn^R^ | This work |
| pVA70 | pJF119EH, P_nar_-(*narGHJI*), Ap^R^ | ([9](#_ENREF_9)) |
| pVA70GFP | pJF119EH, P_nar_-(*narG-egfp,narHJI*), Ap^R^ | ([9](#_ENREF_9)) |
| pSWU19 *sfgfp-linker-frzX* | pSWU19, P_frzX_-(*sfgfp-linker-frzX*) | (66) |
| Name | **Primer sequence (5’ - 3’)** | |
| *nirB*(fwd) | AGCCGTCACCGTCAGCATAAC | |
| *nirB*(rev) | CGTCTTTCACTCGCGCTTCGTAATG | |
| *hcp*(fwd) | CACGGTTTTATTCTTAGCCTGTTAGTG | |
| *hcp*(rev) | TGGCGTGGAGGAAATGGTGTAAG | |
| *norV*(fwd) | ACGGAAAAACTCATCTTTG | |
| *norV*(rev) | GGCAAATTCACCCGCCGTCTGG | |
| *hmp*(fwd) | ATTACCTTCAGGCTACGCAAGGCTTTGGAG | |
| *hmp*(rev) | GAGATTGTCGTACCGGACGACATTG | |
| *nfrA*(fwd) | GAGGAAGATACTGACTAACTC | |
| *nrfA*(rev) | CATGCATACCTTCGGTATCTGG | |
| *fdoG*(fwd) | GAAATTGAGCCAATTCTGGACC | |
| *fdoG*(rev) | GCCACTTCATTCCTGGAAGTC | |
| *sfgfp*(fwd) | AGAAGGCAGAAGCGAAAAAAGAGAGTGAAGAAGGGATATCTAAAGGTGAAGAACTGTTC | |
| ‘*fdnI-sfgfp*(fwd) | AAGAAACACCATCCGCGCTGGTATCGTGAAATCGAGAAGGCAGAAGCGAAAAAAGAG | |
| *kn*(fwd) | GGATGAGCTCTACAAATAAATTCCGGGGATCCGTCGACC | |
| *sfgfp*(rev) | GGTCGACGGATCCCCGGAATTTATTTGTAGAGCTCATCC | |
| *kn*(rev) | CATGAACAAAGCCTACGTTGTCTACAATATGAAAATGGCGCCTTTTACAGTGTAGGCTGGAGCTGCTTC | |
| *fdnI*(fwd) | GGCATTTTGGGTGAAAGGATCG | |
| *fdnI*(rev) | CATTTGAAACTGATGACAAACGC | |
